# He’s just content to sit: a qualitative study of mothers’ perceptions of infant obesity and physical activity

**DOI:** 10.1186/s12889-017-4503-5

**Published:** 2017-06-19

**Authors:** Danae Dinkel, Kailey Snyder, Anastasia Kyvelidou, Victoria Molfese

**Affiliations:** 1 University of Nebraska at Omaha, School of Health and Kinesiology, 6001 Dodge Street, Omaha, NE 68182 USA; 2 University of Nebraska at Omaha, Department of Biomechanics, 6160 University Drive, Omaha, NE 68182 USA; 3 University of Nebraska at Lincoln, College of Education and Human Sciences, 1400 R St Lincoln, Lincoln, NE 68588 USA

**Keywords:** Infant, Physical activity, Qualitative, Obesity

## Abstract

**Background:**

Rates of obesity among children ages zero to five are rapidly increasing. Greater efforts are needed to promote healthy behaviors of young children. Mothers are especially important targets for promoting health as mothers’ views play a vital role in helping their children foster healthy habits from an early age. Research has found parents’ views of infants’ weight may influence their feeding practices; however, limited research has explored mothers’ view of infants’ weight in relation to the promotion of physical activity. Therefore, the purpose of this study was to explore the perceptions of mothers of normal weight infants and overweight infants about their infant’s weight and physical activity.

**Methods:**

Semi-structured interviews were conducted with mothers of normal weight (*n* = 18) and of overweight (*n* = 11) infants (6.5 ± 0.5 month) in a Midwestern city in the United States. A thematic analysis was used to analyze the data.

**Results:**

A majority of mothers thought infants could be overweight. However, no mothers referenced their own infant as overweight. Mothers most commonly noted infants could be overweight only if they were formula fed and/or were overfed, not if they were breastfed. Mothers views were not negatively influenced by others who mentioned that their child was either “big” or “small” and only one mother had been told her infant was overweight. A majority of mothers thought an infant could be physically active. When discussing infant activity, mothers primarily referred to it in terms of general mobility and a few thought activity level was related to a personality characteristic. Mothers intended to promote physical activity in the future either through outdoor play or specific organized activities such as sports. Despite a majority of mothers stating they were currently physically active themselves, only a few talked about interacting with their infant to promote their infant’s physical activity.

**Conclusions:**

Efforts are needed by healthcare professionals and other public health professionals to inform mothers about the dangers of increased weight during infancy as well as the importance of interacting with infants to promote physical activity.

**Electronic supplementary material:**

The online version of this article (doi:10.1186/s12889-017-4503-5) contains supplementary material, which is available to authorized users.

## Background

Rates of obesity among children ages zero to five have increased dramatically all over the world [1]. This is concerning as the first year of a child’s life can make a significant impact on his/her long-term health [2]. Specifically, rapid increases in weight during the first 6 months have been associated with increased risk for obesity at ages 5 and 10 years [3]. Unfortunately, overweight and obesity has a negative impact on a child’s physical and psychological health as well as overall quality of life [4]. For example, research studies have found cardiometabolic risk factors and development of atherosclerosis in obese preschoolers, once thought to only present in adulthood [5, 6]. In addition to these concerning findings, being obese as a child increases one’s risk of adult obesity, which is associated with a variety of chronic health conditions. The obesity epidemic has had severe economic impact for countries all over the world ($73.8 billion in US, $10.9 billion in Germany) [7]. Greater efforts are needed to prevent and treat infants (<12 months of age) who are at-risk for being overweight or obese (defined as having a weight-for-length (WFL) z-score greater than two standard deviation above the World Health Organizations (WHO) standards) [4, 7].

Reducing the prevalence of overweight and obesity rates is no small task as a multitude of factors influence a child’s weight status such as genetic/biological, behavioral, and environmental factors as well as an individual’s culture and socioeconomic status [4]. One of the recommended strategies to combat childhood obesity by the WHO is to “provide guidance on, and support for, healthy diet, sleep and physical activity in early childhood to ensure children grow appropriately and develop healthy habits” [4, pg. 26]. Within a child’s first year (i.e., infancy), parents play an obvious role in supporting and developing healthy habits. However, there is evidence that many parents fail to identify their infant as being overweight or obese because they prefer “chubbier” infants and health professionals (i.e., pediatricians) who parents look to for advice may also not acknowledge children’s overweight or obesity status [8–19].

### Parental preference for larger infants

Several studies have found parents prefer fatter infants, especially those parents whose infants are heavier [14, 16]. For example, a survey conducted by Laraway and colleagues [14] found that almost a quarter of parents of 6–27 month olds preferred their infant’s weight to be in the highest quartile on the WFL growth chart. This may be due to parent’s belief that higher percentiles equate to superior standings (i.e., academic achievement scores) [14]. Redsell and colleagues [16] found similar results in their qualitative study of parents of infants in the United Kingdom. However, the theme of a preference for heavier infants was only found in parents who were overweight or obese themselves. Additionally, parents’ perceptions were influenced by cultural or individual preferences such as what friends and family thought and advice received from previous generations (e.g., grandparents).

Conversely, parents may not recognize their infant is overweight. Jimenez-Cruz and colleagues [13] found that 83% of mothers from Mexico with 5 to 24-month olds who were overweight or obese underestimated their infant’s weight. Thus, parents may need guidance on recognizing overweight and obese infants and guidance on why and how to promote healthy behaviors as preventative efforts against childhood obesity beginning in infancy [16]. Additional in-depth exploration is needed to better understand parental perceptions of infants’ weight (normal weight, overweight) and how these perceptions may impact promotion of healthy behaviors [14].

### Fostering healthy habits

Parents are especially important targets for promoting healthy behaviors as they play a vital role in helping their children foster healthy habits from an early age [4, 20, 21]. Research in this area has primarily focused on the influence of parental attitudes in the development of children’s eating practices [13, 14, 21–26]. Another critical habit that parents begin to influence during infancy is the adoption of healthy physical activity practices. In general, it is recognized that parents play a critical role in their infant’s motor development as they make decisions on how much an infant is held, the time spent in restrictive devices (i.e., carriers, car seats, swings), the time given for play on their stomach, and infant physical activity in general [27]. To promote infants’ health and motor development it is recommended that infants take part in daily developmentally appropriate activities (e.g., rolling, crawling) in open and safe play areas in which infants have the ability to take part in free movement with appropriate toys (e.g., rattles, balls). Additionally, guidelines suggest promoting parent-infant interaction and limiting time spent in restrictive devices (e.g., swings, car seats) [28]. Understanding these perceptions is important as research has shown that a child’s movement and development of gross and fine motor skills is critical for muscle strengthening and participation in physical activity later in life [29, 30]. However, research has found that few parents view physical activity as a top priority for infants and few are concerned about practices that may limit their infant or young child’s activity, such as stroller use [16, 31, 32].

Interestingly, studies have found parents perceive high infant activity levels as problematic due to concerns of inappropriate behaviors in certain situations, discipline concerns, or the physical demands of having to chase a child around [32–34]. A negative view of activity in infancy may lead to parents wishing their infant were less active which could increase the promotion of more sedentary activities. For example, Thompson and colleagues [34] determined infants of low-income African American mothers’ who were perceived as being more active had higher odds of TV viewing. More research is needed with different socio-economic classes and in younger samples to gain a fuller picture of mothers’ perceptions of infants’ physical activity. Additionally, few studies have fully explored mothers’ of both normal weight and overweight infants’ perception of physical activity or mothers’ awareness of physical activity guidelines [13, 16, 32].

### Physicians’ recognition of obesity in infants

Within the United States, pediatricians serve as the cornerstone of children’s healthcare. While little research has examined pediatrician’s discussion of weight specifically with the parents of infants, several studies have found that often pediatricians do not acknowledge older children’s weight or are uncomfortable discussing with parents that a child is overweight or obese [8, 10, 12]. This is concerning as the American Academy of Pediatrics recommends pediatricians see children 14 times prior to their fifth birthday, providing ample opportunities to discuss weight concerns with parents [35]. The lack of acknowledgement from pediatric physicians may also contribute to parents’ lack of concern about their child’s weight [11]. However, more research is needed regarding parent’s perception of pediatrician’s acknowledgement of weight, especially in the first year.

One theory that has been utilized in health behavior studies is the Social Cognitive Theory [36]. The Social Cognitive Theory posits that behavior can be explained based on the interaction between personal cognitive factors (self-efficacy, collective efficacy, outcome expectations, knowledge), socioenvironmental factors (observational learning, normative beliefs, social support, opportunities and barriers), and behavioral factors (behavioral skills, intentions, reinforcement). A better understanding of these concepts in relation to parents perceptions of infant weight and physical activity could help inform the development of interventions designed to improve infant health.

Therefore, the central research question was: How do mothers’ of normal weight infants (NWI) and overweight infants (OWI) perceive their infant’s weight and physical activity? The sub-questions are as follows: What beliefs of the mother influence these perceptions? What external factors influence these perceptions? These findings are needed to increase our understanding of parents’ current knowledge and use of physical activity-related practices in infancy. This information may be useful for developing strategies to enable parents to engage with their infants in physical activities, especially those at-risk for obesity.

## Methods

### Participants and recruitment

Parents were recruited to participate in a larger study examining the physical activity and postural control of NWI and OWI in a Midwestern city in the United States. Full details of the study are presented elsewhere [37]. A purposeful sampling strategy was used to recruit primary caregivers consisting of NWI and OWI [38]. Recruitment occurred via flyers in local baby friendly businesses (e.g., chiropractic offices), women & infant clinics (WIC), social media (e.g., Facebook), physician referral, and word of mouth (e.g., family/friend recommendations). Inclusion criteria consisted of being the primary caregiver of a typically developing infant <3 months of age. Exclusion criteria included having an infant with a birth defect or congenital abnormality, having been born preterm (<37 weeks), being classified as underweight (WFL z-score less than two standard deviations below the median), or any other diagnosed medical condition that might affect brain development, visual, auditory or motor impairment. Eligible participants were contacted via text or e-mail to ensure they met the inclusion/exclusion criteria and to confirm interest in participation. Upon response, participants were scheduled for an initial intake appointment. Recruitment was halted once participation numbers were reached (*n* = 36). Participants completed a written consent form if they agreed to participate in the study.

A total of 51 mothers indicated an interest in the study by contacting researchers. Twenty-two were either excluded prior to their initial visit (*n* = 2 infants underweight, *n* = 11 infant too old, *n* = 2 diagnosed health condition, *n* = 2 born premature) or dropped out after the first visit (*n* = 5) due to lack of time or moving out of the area. Thus, 29 mothers completed the study. The majority of mothers were White (93.1%), between the ages of 20–30 (55.2%), and worked full-time (44.8%) or were homemakers (44.8%). Three-fourths (75.8%) of mothers were overweight or obese and a little over a third of the infants were overweight (37.9%) (Table 1). Of the 29 infants, 68.9% were exclusively breastfed for at least 6 months. At the time of the interviews infants were 6.5 months old (± 0.5).

**Table 1:** Participant demographics

Characteristics	Mothers *n* = 29	Infants *n* = 29
Weight		
Normal weight	7 (24.1)	18 (62.1)
Overweight	8 (27.6)	11 (37.9)
Obese	14 (48.3)	
Race/Ethnicity		
White	27 (93.1)	22 (75.9)
Asian	1 (3.4)	1 (3.4)
Hispanic or Latino	1 (3.4)	1 (3.4)
More than one race/ethnicity		5 (17.2)
Age (years)		
20–30	16 (55.2)	
31–40	13 (44.8)	
Mean (SD)	30.7 (±3.5)	
Education		
High School	2 (6.9)	
Some College	8 (27.6)	
Bachelor’s	9 (31.0)	
Graduate Degree	10 (34.5)	
Employment		
Full-Time	12 (41.4)	
Homemaker	12 (41.4)	
Other	5 (17.2)	
Household Income		
$21 k-$40 k	4 (13.9)	
$41 k-$60 k	5 (17.2)	
$61 k-$80 k	15 (51.7)	
$81 k+	5 (17.2)	
Exclusive Breastfeeding		
≥ 6 mo. of age	20 (68.9)	
≤ 6 mo. of age	9 (31.1)	

### Data collection

As part of the overall study, participants completed three visits to the University’s motor development lab: at three months of age, the onset of sitting (~5 months of age), and one-month post onset of sitting (~6 months of age). Infants’ weight was measured to the nearest 10 g via a Detecto Pediatric scale and length was measured to the nearest .1 cm via a SECA infantometer in order to calculate their WFL z-score [39, 40]. Infants were classified as overweight (*n* = 11) if their WFL Z-score was above two standard deviations of the WHO’s standards at one or more lab visits; otherwise they were classified as normal weight (*n* = 18). Mothers completed a short demographic survey at each visit including items such as breastfeeding duration and their own current weight.

Semi-structured interview questions were developed based on a previous study exploring parents’ beliefs about infant weight, growth and feeding behaviors [16]. Questions were modified to more specifically explore infants’ physical activity and motor skill development (for the full list of interview questions, please see the Additional file 1). Pilot testing was conducted with the first two interviews to determine any weaknesses or flaws in the interview protocol. Minor revisions were completed prior to conducting the remaining interviews. The interview guide included a combination of closed and open-ended questions. Questions pertained to: 1) caregivers’ perception of infant weight, 2) knowledge of developmental milestones, 3) typical activities for their infant, and 4) future physical activity promotion efforts for their child. While the guide was designed to discuss these topics, interviewers were allowed flexibility in which to explore additional topics that may have arisen during discussions [41].

Semi-structured interviews were conducted with mothers (*n* = 29) from May 2015–March 2016. All interviews were conducted by one of two trained research personnel within the University’s motor development lab. The interviews occurred at the third visit at a time convenient to the mother and infant. Mothers received $25 per visit for participating in the study. Interview duration ranged from 15 to 34 min with an average duration of 22 min. All interviews were audio recorded and transcribed verbatim by research personnel experienced in transcription. All participants were given identifying codes and names were not associated with the transcripts. The word count for interviews ranged from 802 to 3172 words with an average of 1834 words. Interview transcripts were reviewed by the researchers who conducted the interviews for accuracy before data analysis.

### Data analysis

Two trained qualitative researchers (DD, KS) conducted a thematic analysis following the methodology outlined by Braun and Clarke [42]. First, both researchers read through each of the transcripts. Next, they independently coded the first two interviews, and then met to compare codes, reach consensus, and develop a codebook. The researchers then re-coded the initial interviews and coded the remaining interviews. The researchers met again to compare all coded data and discuss any additional issues that arose during coding (e.g., addition of other codes). Any discrepancies in coding were discussed with a third researcher trained in qualitative methods in order to reach a consensus on all coded data. Codes were categorized into overall themes guided by the two main purposes of our study: examining perceptions of weight and physical activity and the researchers then independently revisited the coding to ensure the themes fully reflected the findings within the data. Finally, the researchers met one last time to finalize themes. Data were validated through the process of peer debriefing which consists of discussion of the findings with impartial colleagues [38, 43].

## Results

The results are organized into two primary themes: perceptions of infant weight and perceptions of physical activity. Quotations from mothers as well as whether they were the mother of a NWI or OWI is noted to help confirm and explain findings.

### Perceptions of infant weight

#### Infants can be overweight

A majority of mothers thought that infants could be overweight. However, many mothers were not definitive in their answers prefacing their answers with “I guess” or “I think it could be possible” without actual reference to their own infant or an infant they knew personally as being overweight. None of the mothers referenced their own infant as overweight. When discussing if an infant could be overweight, mothers most commonly noted only infants who were formula fed and/or were overfed either by a bottle or because they started solid foods too early could be overweight. As a majority of the mothers in the study had breastfed, there was a strong belief that if infants were breastfed it was not possible for them to be overweight. For example, one mother stated, “I think it’s impossible for a breastfed baby to be overweight. Um, I suppose a formula fed baby could be overfed if that parent, or care provider was constantly using that as the only tool to use to calm, to meet their needs…but I don’t think that it’s possible with a breastfed baby” (mother of NWI). Another mother mentioned “I mean, especially breastfed babies are probably not as at risk but, you know, I mean if they…. bottle feed, you know, you get the sugar in there. Especially if you start giving them solids early you can get a little, I mean…there is gestational diabetes babies. Like, those things are fat. You know, you have a 14-pound newborn…. that’s a fat baby” (mother of OWI).

Conversely, a smaller portion of mothers did not believe it was possible for an infant to be overweight. Their reasoning was similar to those who thought they could be, due to exclusive breastfeeding. As one mother noted: “At this age? No. (Laughter)…I just don’t think at 8 months, especially breastfed…I just don’t think they can…I don’t think they’re eating enough of foods that make you overweight yet” (mother of OWI). Prevalent in both those who did and did not think infants were overweight was an underlying belief that at this age it was too soon to classify infants in a category with such negative connotations.

#### Mothers views are not negatively influenced by others

Mothers rarely mentioned that others within their social network (pediatrician, family, friends) negatively impacted their beliefs about their infants’ weight. This was primarily due to either a lack of discussion about weight or lack of care of what others thought. Specifically, only one mother reported being told their infant was overweight or at-risk of being overweight by a pediatrician. This mother of an OWI revealed her pediatrician had mentioned a concern about her infant’s weight but then the provider changed their mind. The parent noted:

Well, when she was little they told me that she was obese and it, I mean obviously you don’t like to hear that. Then I was at the pediatrician by myself with her and I told them, I told her about her Dad’s side [big] and they were like oh never mind. So I was like ok so you need to, they need to do a lot of background. Before they were just like well she is just obese. She’s a big girl, she always has been...

Almost all mothers mentioned that other people had commented on the size of their baby primarily as either being big or small. Most mothers appeared to view these comments positively and that it was common in society to make comments about the size of a child particularly in regards to how they were growing. Over half of all mothers discussed that family, friends, and complete strangers had commented that their child was “big”. Interestingly, only a portion of these mothers were a mother of an OWI. One mother mentioned that people commented “Just that he’s healthy, chubby, big” (mother of OWI). As one mother mentioned, “they always say he’s large but we know he’s about average so it’s fine” (Mother of NWI). Another mother of an OWI mentioned others had stated “Just that he’s growing fast, his chunky thighs, but I like those.”

Several mothers discussed how people made comments that their infant was small for their age. Almost all of the mothers who commented that others said their child was small were mothers of NWI; however, one mothers’ infant was overweight. This mother stated “They say she is so small all of the time…but I think it’s because her head is little.” Similar to mothers who reported people mentioning the larger size of their infants, mothers who mentioned the smaller size also viewed this as positive and were aware of their infant’s small stature. Of the few mothers who appeared to perceive comments about the size of their infant negatively, all felt that others did not know the complete picture of the health of their infant and were not “bothered too much” by these comments. As one mother of an OWI noted “Sometimes they can say that he’s hefty or husky which to me they just don’t know what they are looking for…they can be a little buggy.”

### Perceptions of infant physical activity

#### Infants can be physically active

A majority of mothers thought an infant could be physically active. When mothers described how their infants took part in activity, they primarily referred to it in terms of general mobility. Interestingly a few mothers who discussed general mobility as the method of infant activity mentioned they thought this was related to a personality characteristic or not being in restrictive devices. For example, a mother of an OWI stated “I think some babies just like to lay and some babies are really…wiggly and moving their arms and kicking and just moving more…definitely some babies are more chill and just like to relax.” Another mother saw these differences within two of her children as she discussed “…like my older child moved a lot more than he does…he’s just content to just sit there” (mother of NWI). A few mothers felt that infants were naturally inclined to be active unless factors in the environment prohibited this activity. For example, a mother of a NWI stated, “Like, if they’re restricted to a play pen or one of those exersaucers or things like that too much. That would definitely limit their ability to burn off energy.”

Others referred to physical activity in terms of it being linked to their infant’s specific movements (rolling, crawling, walking). One mother of a NWI mentioned “…being able to like push himself up when he’s on his tummy” and another mother stated “kicking and rolling and running and crawling.” A handful of mothers also mentioned their infant was physically activity only in terms of playing with toys such as jumpers as one mother described her child was active when “actively jumping in their jumper and not just like standing there propped up against [something]” (mother of a NWI).

#### Intention for future promotion of physical activity

When asked about what type of activities they would promote to help their kids be active as they continued to grow, the most common answers included promotion of playing outside in general or organized activities such as sports. Interestingly mothers of OWIs more often discussed playing outside while mothers of NWIs more often mentioned sports and organized classes. One mother of an OWI stated “Um, you know, going to the park and going on walks and bike rides.” While a mother of a NWI said “now that he’s um, old enough to do swim lessons we’ll probably do that and then, just playing outside all the time or playing sports mostly, is what we do with his older brother too.” Only half of mothers specifically mentioned they would promote activity on a daily basis.

#### Family and mothers’ impact on physical activity

Almost half of mothers mentioned the potential positive impact their family would have on their infants’ physical activity levels. This was either due to the parents, siblings and/or the entire family being active. As one mother mentioned, “Um, his mom and dad are both very physically active. Um, his older brother is too so it’s just kind of our lifestyle” (mother of NWI). Another mother of an OWI stated, “We all workout. We have dogs. Yep, going for walks. Um, I workout. I do like the home workout videos. Um, and so, as he’s been little I, I incorporate him into those so I feel like as he gets older he’ll want to do those with us.” When discussing an infant’s current physical activity though, only a few talked about the importance of infants interacting with others during this time. For example, one mother mentioned the importance of being “physically active by like being on the floor, encouraging them to roll, encouraging them to crawl” (mother of NWI).

A majority of mothers appeared to be role modelling physical activity as they stated they were currently physically active with over half of mothers being active four or more days per week. The most common activities mothers participated in were walking or some type of cross-training/strength activity. One mother of an OWI stated “Um, just, I walk. Um, every morning. Um, that’s about it and chasing kids.” Another mother discussed “I lift, crossfit, and run, and walk. I have to. It is just when I, it’s my release, it’s my one hour alone, every day” (mother of NWI). Despite mothers talking about their own physical activity levels, only a few mentioned the importance of parent modeling in the promotion of healthy behaviors and all but one of these mothers had an OWI. As the mother of an OWI stated, “I mean a lot of it is what they see you doing in the household. What you are feeding them, what they see you eating, the things you are cooking, drinking, those kinds of things. So, um, a lot of it is, you know, what you are doing.” However, none of the mothers specifically mentioned role modeling in relation to their own physical activity levels.

## Discussion

This study aimed to understand how mothers of NWI and OWI perceive their infant’s weight and physical activity. A general theme was mothers’ perceptions of infant weight. Although most thought infants could be overweight, primarily due to overfeeding and/or using formula, no caregivers included their own infant in this description. Many thought that breastfed infants could not be overweight. This could be a normative belief of their society, a majority of whom exclusively breastfed their infants for six months or longer, which may be leading women to associate formula feeding as a negative behavior thus leading to negative health outcomes such as obesity. Interestingly, there is evidence that breastfeeding is associated with a lower prevalence of obesity later in life and has been promoted as an obesity prevention strategy [44]. However, more information regarding parental overfeeding and its link to infant weight for both breast and formula fed infants is needed [26]. Breastfeeding alone does not completely buffer an overweight child from long-term risk of obesity. Since knowledge is a precursor to health behavior change, making parents aware of other risk factors during infancy such as rapid increases in weight could be important for obesity prevention efforts [36, 45]. Additionally, future research could explore the social norms and beliefs regarding weight in breastfeeding mothers.

Relatedly a person’s cultural norms and beliefs (normative beliefs) can also greatly influence what is perceived to be socially acceptable, thus impacting mothers parenting practices [36]. Specifically, other researchers have found family members, especially grandparents, can have a large influence on an infant’s feeding practices [46, 47]. As such, family members or comments by others about infants could also influence parents’ perception of their infant’s weight. Yet regardless of infant size, few mothers viewed these comments about the size of their infant as negative. For those mothers who had heard comments that their child was “big”, this may reflect a similar finding from other studies that mothers prefer “fatter” infants and/or this is the cultural norm of how one talks about infants [14, 16]. Redsell and colleagues [16] only found a preference for larger infants from parents who were overweight themselves. Overall, in this study, comments from others did not appear to be a factor in parents’ perceptions of their infants’ weight.

Our findings appear to be consistent with previous research in that pediatricians may not be having conversations regarding infant weight [8, 10, 12]. Only one mother could recall her pediatrician addressing her infant’s weight and after learning of a family history of large size the pediatrician recanted their concern. As 11 of the infants in this study were overweight it is noteworthy that so few pediatricians had addressed this subject with parents. Future research should assess pediatricians’ recollection of discussing weight with parents of infants as well as to determine what pediatricians take in to consideration when deciding to discuss concerns about weight and how to promote healthy eating and physical activity habits during infancy.

In regards to mothers’ behavior, their interview responses reflected their awareness of some general physical activity guidelines but few reported engaging in behaviors to promote physical activity such as interacting with their child in physical activities. This is concerning as this is a key aspect of the physical activity recommendations and a critical need of healthy early development [48]. The lack of discussion of interacting with their infant may be a result of mothers’ perceptions that infants are naturally active and are not in need of maternal guidance and interaction [32, 49] or due to the views of some mothers in our study and others who thought the activity levels of infants was due to personality characteristics [50, 51]. The lack of reported interaction could also be due to mother’s lack of self-efficacy for promoting infants’ activity. Evidence suggests that many young children do not achieve recommended levels of physical activity [52, 53]. Future research studies could examine parent’s self-efficacy for interacting with their and providing physical activity for their infants. Regardless, efforts to increase parents’ knowledge of the importance of physical activity and to enhance their self-efficacy for engaging in physical activities with infants is warranted [32]. Offering low or no-cost infant/parent play classes at a hospital or community center for parents to obtain knowledge and mastery experiences could be a viable strategy to verbally encourage mothers to interact during this play time. Relatedly, parents and other caregivers (e.g., family members, childcare providers) should be made aware of the potential negative impact of infant sedentary behaviors (i.e., sitting in restrictive devices, watching television). While research is still needed to examine the impact of these behaviors in infancy, given the impact sedentary behaviors has had on older populations the public health and healthcare community should not wait for evidence to mount and be proactive in their promotion of healthy physical behaviors in infancy.

Interestingly, mothers discussed playing outside or organized classes such as sports as future physical activity promotion efforts. Outdoor play offers numerous benefits to health including motor development and mental health [54, 55]. Further, participation in organized sport could provide opportunities to increase behavioral skills for life-long participation in physical activity and participation is associated with a lower body mass index (BMI) [56, 57]. The role of weight in the early development of these healthy physical activity habits is not fully understood. Some research suggests adiposity (e.g., central adiposity, skinfold assessments) is associated with delays in the achievement of motor milestones in infancy (e.g., rolling over, sitting up, walking) which could deter future participation in general physical activity and sport; however, more research is needed [58].

Regardless of the type of physical activity, it is important to note that all mothers intended to promote physical activity. These opportunities provide avenues for mastery experiences and observational learning with peers and family members, which could lead to improved physical activity. Further the promotion of these activities could provide instrumental (i.e., taking child to sport practice) or emotional (i.e., verbally encouragement) support which is also linked to improved physical activity levels [36, 59, 60]. It is important to note that while not specifically asked, these mothers did appear to have access to opportunities for their child to be physically active based on their own physical activities. Lack of access to appropriate physical activity environments due to issues such as lack of safety, sidewalks or open space has been a consistent barrier to physical activity found in the literature [61]. Increased opportunities for activity through avenues such as a safe environment or access to a park are important to the development of healthy behaviors [36, 61]. More research is needed to explore if parents have taken these factors into consideration when considering future physical activity promotion efforts.

Despite the fact that over half of mothers were currently active for four or more days/week, few mothers mentioned the importance of role modeling and none discussed actually participating in physical activity with their infants as a way of role modeling. Role modeling can provide infants and young children opportunities to learn from their mothers how to be physically active which can also lead to later increases in their own self-efficacy for physical activity [36]. Several studies report the important influence parental activity and support for physical activity can have on children’s activity levels [32, 62–64]. Specifically, Hnatiuk and colleages [65] found that the amount of time an infant spent being physically active with their mother at 9-months of age predicted physical activity levels at 19-months of age. As some mothers in this study mentioned they purposefully were active alone as a “release”, encouraging mothers to spend additional time with their infants being active may be important for infants to observe and learn about physical activity to establish long-term physical activity habits.

Additionally, multiple mothers reported that the potential positive impact of their family and/or siblings could have to promote the infant’s future physical activity. When families are physically active together this could promote collective efficacy for physical activity which could lead to improved physical activity levels for the entire family. Further, this could contribute to a child’s normative beliefs about physical activity early on, believing that physical activity is a socially acceptable behavior [36]. Previous findings show older siblings activity is positively associated with the younger siblings [66, 67]. However, if physical activity is limited just to siblings, parents may believe physical activity is a natural inclination of all children [32]. Thus, parents might not see the value of older siblings’ role-modeling positive physical activity behaviors.

Importantly, collective efficacy may not be limited to families. A previous research study found an association between time spent with children of the same age at 4-months of age and physical activity at 19-months of age [65]. Future physical activity promotion efforts should focus on the importance of family-based approaches and play groups with respect to both role modeling and engaging in physical activity opportunities together. Further research should also examine the impact of mother/infant active play groups on infant and early childhood physical activity.

### Strengths and limitations

This study is strengthened by exploring the perceptions of mothers of both NWI and OWI about infant obesity and physical activity. However, due to the sample characteristics (e.g., participants resided in the Midwest, included only mothers, most exclusively breastfed for six months or longer, and reported middle to high-income levels), the findings may not be readily generalizable to other populations. Future research studies should explore these topics in different regions, with both mothers and fathers, with a variety of different feeding strategies, and broader socioeconomic characteristics. Other limitations included the classification of infants as overweight if they were deemed to be overweight at any of the three appointments and the study design in which interviews only occurred at the third visit. Future research should explore differences in parents’ perception of OWI and NWI based on consistency of classifications at all time points with a larger sample size in order to provide a more systematic comparison to examine differences. A further limitation is the length of the interview (average length 22 min). Given that infants were with mothers during the interviews and they had taken part in additional measures for the overall study, the research team determined that a shortened interview guide could still provide detailed information and lessen participant burden. An additional limitation is that the findings were analyzed by researchers involved in the study, which could have biased the findings. While the researchers took part in validation strategies including reviewing the findings with an external researcher, including an external researcher in the preliminary analysis could have further strengthened the findings. Finally, it is important to note that societal view of childhood obesity and concern about obesity prevention beginning in infancy and the importance of physical activity are relatively recent targets of concern in early childhood. Thus, the pediatricians’ role in providing information to parents regarding infant obesity and physical activity and the sources of information available to parents on these topics are both changing, offering rich opportunities for further research.

## Conclusions

Overall, this study shows that most mothers thought infants could be overweight, primarily due to overfeeding and/or using formula, yet did not identify their own infant as overweight. In addition, mothers thought infants could be active but rarely mentioned their own interaction with infants as a part of promoting physical activity with their child. Efforts are needed to inform mothers about the dangers of overfeeding with both breast and formula fed infants as well as the importance of being active with their infants. Without prompting, mothers discussed several topics related to key factors of Social Cognitive Theory that could lead to long-term healthy physical activity habits for infants such as family social support and collective efficacy for activity. However, issues such as normative beliefs regarding ability of breastfed infants to be overweight and mothers’ potential lack of self-efficacy for promoting infant activity could deter infant physical activity promotion efforts. Interventions grounded in Social Cognitive Theory, could lead to improved infant health. For example, interventions tailored towards increasing parents’ knowledge of risks associated with infant obesity and the importance of infant physical activity as well as ensuring equitable opportunities for infants and families to take part in physical activity could help to alter societal norms and expectations as well as enhance mothers’ self-efficacy regarding engaging in activity with their infants. To best understand the influence of physical activity promotion and interaction between mothers and infants on physical activity, motor skill development, and obesity, a larger-scale study is needed. Additionally, future research studies should examine the influence of fathers as well as cultural and ethnic beliefs about obesity on infant weight and physical activity behaviors.

## Additional file

Additional file 1: Interview Questions. Semi-structured interview questions. The full list of interview questions. (DOCX 13 kb)

## Abbreviations

NWI: Normal weight infant
OWI: Overweight infant
