# Supplementary material for: He’s just content to sit: a qualitative study of mothers’ perceptions of infant obesity and physical activity
Source: BMC Public Health. 2017 Jun 19;17:585. doi: 10.1186/s12889-017-4503-5 (PMC5477242; doi:10.1186/s12889-017-4503-5)
Supplement: Additional file 1: — Interview Questions. Semi-structured interview questions. The full list of interview questions. (DOCX 13 kb) [file 12889_2017_4503_MOESM1_ESM.docx]

| Semi-Structured Interview Questions | |
| --- | --- |
| Interview Question |  |
| 1. When you hear the term infant motor skill development what comes to mind? 2. When you think of infant physical activity what comes to mind? 3. In the next few years what types of activities do you plan on promoting with your infant? 4. What type of play materials do you use most frequently? 5. Describe a typical week and weekend day for your infant. 6. When you think of the term infant milestones, what comes to mind? 7. Has anyone ever referred to your child as delayed or advanced for their age? 8. Can you describe your baby’s size at birth and his/her growth over the months? 9. Has your baby been identified as “at risk” of being overweight by a health professional? 10. Is it possible for a baby to be overweight? Why or why not? 11. Do you know of any ways to prevent childhood obesity? 12. If a baby was putting on too much weight at what point do you think a health professional should talk to the parents? 13. Are you currently physically active in your free time?   a. If yes,  i. How often? Times/week, amount of time (30 min/day)  ii. What activities?  b. If yes, why do you participate in physical activity?  c. If not, why do you not participate in physical activity?  i. What is needed to help you participate in physical activity? |  |
